# Union Makes Strength: A Worldwide Collaborative Genetic and Clinical Study to Provide a Comprehensive Survey of RD3 Mutations and Delineate the Associated Phenotype

**DOI:** 10.1371/journal.pone.0051622

**Published:** 2013-01-07

**Authors:** Isabelle Perrault, Alejandro Estrada-Cuzcano, Irma Lopez, Susanne Kohl, Shiqiang Li, Francesco Testa, Renate Zekveld-Vroon, Xia Wang, Esther Pomares, Jean Andorf, Nisrine Aboussair, Sandro Banfi, Nathalie Delphin, Anneke I. den Hollander, Catherine Edelson, Ralph Florijn, Marc Jean-Pierre, Corinne Leowski, Andre Megarbane, Cristina Villanueva, Blanca Flores, Arnold Munnich, Huanan Ren, Ditta Zobor, Arthur Bergen, Rui Chen, Frans P. M. Cremers, Roser Gonzalez-Duarte, Robert K. Koenekoop, Francesca Simonelli, Edwin Stone, Bernd Wissinger, Qingjiong Zhang, Josseline Kaplan, Jean-Michel Rozet

**Affiliations:** 1 Unité de Recherches Génétique et Epigénétique des Maladies Métaboliques, Neurosensorielles et du Développement (INSERM U781)- Université Paris Descartes- Fondation IMAGINE, Paris, France; 2 Department of Human genetics, Radboud University Nijmegen Medical Centre, Nijmegen, The Netherlands; 3 McGill Ocular Genetics Laboratory, Montreal Children's Hospital, McGill University Health Centre, Montreal, Canada; 4 University Eye Hospital, Institute for Ophthalmic Research, Tübingen University, Tübingen, Germany; 5 State Key Laboratory of Ophthalmology, Zhongshan Ophthalmic Center, Sun Yatsen University, Guangzhou, China; 6 Department of Ophthalmology, Second University of Naples, Naples, Italy; 7 The Netherlands Institute for Neuroscience (NIN-KNAW), Amsterdam, The Netherlands; 8 Department of Molecular and Human Genetics, Baylor College of Medecine, Houston, Texas, United States of America; 9 Faculty of Biology, Department of Genetics, University of Barcelona, Barcelona, Spain; 10 Department of Ophthalmology and Visual Sciences, The University of Iowa Carver College of Medecine, Iowa City, Iowa, United States of America; 11 Service de Génétique CHU Mohammed VI, Faculté de Médecine et de Pharmacie, Université Caddi Ayyed, Marrakech, Morocco; 12 Telethon Institute of Genetics and Medecine (TIGEM), Naples, Italy; 13 Medical Genetics, Department of General Pathology, Second University of Naples, Naples, Italy; 14 Department of Ophthalmology, Radboud University Nijmegen Medical Centre, Nijmegen, The Netherlands; 15 Ophtalmo-pédiatrie, Fondation Rothschild, Paris, France; 16 Institut Cochin, Université Paris Descartes, Paris, France; 17 Institut National des Jeunes Aveugles, Paris, France; 18 Service de Génétique Médicale, Université Saint Joseph, Beyrouth, Lebanon; 19 Servicio de Génética, Asociacion Para Evitar La Ceguera en Mexico, Mexico City, Mexico; Odense University Hospital, Denmark

## Abstract

Leber congenital amaurosis (LCA) is the earliest and most severe retinal degeneration (RD), and the most common cause of incurable blindness diagnosed in children. It is occasionally the presenting symptom of multisystemic ciliopathies which diagnosis will require a specific care of patients. Nineteen LCA genes are currently identified and three of them account for both non-syndromic and syndromic forms of the disease. *RD3* (*LCA12*) was implicated as a LCA gene based on the identification of homozygous truncating mutations in two LCA families despite the screening of large cohorts of patients. Here we provide a comprehensive survey of *RD3* mutations and of their clinical expression through the screening of a cohort of 852 patients originating worldwide affected with LCA or early-onset and severe RD. We identified three *RD3* mutations in seven unrelated consanguineous LCA families - *i.e.*, a 2 bp deletion and two nonsense mutations – predicted to cause complete loss of function. Five families originating from the Southern Shores of the Mediterranean segregated a similar mutation (c.112C>T, p.R38*) suggesting that this change may have resulted from an ancient founder effect. Considering the low frequency of *RD3* carriers, the recurrence risk for LCA in non-consanguineous unions is negligible for both heterozygote and homozygote *RD3* individuals. The *LCA12* phenotype in our patients is highly similar to those of patients with mutant photoreceptor-specific guanylate cyclase *(GUCY2D/LCA1*). This observation is consistent with the report of the role of RD3 in trafficking of GUCYs and gives further support to a common mechanism of photoreceptor degeneration in *LCA12* and *LCA1*, *i.e.*, inability to increase cytoplasmic cGMP concentration in outer segments and thus to recover the dark-state. Similar to *LCA1*, *LCA12* patients have no extraocular symptoms despite complete inactivation of both *RD3* alleles, supporting the view that extraocular investigations in LCA infants with *RD3* mutations should be avoided.

## Introduction

Leber congenital amaurosis (LCA, MIM# 204000) is a group of retinal dystrophies, which represents the most common cause of blindness in childhood (10–18%) [1], [2]. This disease is responsible for blindness or profound visual deficiency at birth or in the first months of life. Visual outcome ranges from a severe stationary cone-rod disease with poor visual function (VA≤light perception; type I) to a progressive rod-cone dystrophy with measurable visual acuity in the first decade of life (20/200≤VA≤60/200; type II) [3]. Currently, 19 disease-causing genes have been mapped and/or identified that account for 65–70% of the cases in ethnically mixed populations [1], [2]: *GUCY2D* (*LCA1*) [4], *RPE65* (*LCA2*) [5], *SPATA7* (*LCA3*) [6], *AIPL1* (*LCA4*) [7], *LCA5* (*LCA5*) [8], *RPGRIP1* (*LCA6*) [9], [10], *CRX* (*LCA7*) [11], [12], *CRB1* (*LCA8*) [13], *NMNAT1* (*LCA9*) [14]–[18], *CEP290* (*LCA10*) [19], *IMPDH1* (*LCA11*) [20], *RD3* (*LCA12*) [21], *RDH12* (*LCA13*) [22], [23], *LRAT* (*LCA14*) [24], *TULP1 (LCA15*) [25], *KCNJ13* (LCA16) [26], *IQCB1* [27], [28] and *MERTK* [29]. Typically, LCA is an autosomal recessive condition. Yet, some rare LCA cases have been ascribed to dominant *CRX* [11], [30]–[31] and *IMPDH1* mutations [20].

The implication of LCA genes in retinal dystrophies of later-onset is not uncommon (*GUCY2D*, *RPE65*, *CRB1, TULP1, CRX, RPGRIP1, AIPL1*) [1], [2]. Though less frequently, LCA genes may be involved in severe and sometimes embryonically lethal multisystemic conditions affecting the central nervous system, kidneys, bones and/or the heart in addition to the retina (*CEP290*, *IQCB1*) [32]–[33].

The clinical, genetic and molecular heterogeneity of LCA make elucidating genotype-phenotype correlations a major medical issue to predict the ophthalmologic course of the disease, to allow tailor-made surveillance of extraocular systems and to direct genetic studies. Significant genotype-phenotype correlations have been reported for most LCA genes; for recent reviews see [1], [34] but not *RD3* which mutations are amongst the rarest causes of the disease with two homozygous mutations reported in two consanguineous families [21], [35].

Here, we report the results of an international study aimed at delineating the clinical and molecular spectrum of *RD3* mutations in retinal dystrophies.

## Patients and Methods

### Patients and Controls

A total of 852 unrelated patients ascertained in -or addressed to- 10 Centers in Europe (France, Germany, Italy, the Netherlands and Spain), North-America (The United States and Canada) and China were considered in this study including 574 LCA probands who have not had disease-causing mutations identified in the known LCA genes, 96 probands affected with autosomal recessive early-onset severe retinal dystrophies (EOSRD), 150 with autosomal recessive retinitis pigmentosa (RP) and 32 with other retinal dystrophies. The ethnicity of patients and diagnoses are shown in Table 1. Written consents were obtained from participants or legally authorized representatives at each participating Center. The study was conducted at all sites in strict adherence to the tenets of the Declaration of Helsinki and was approved by the respective National Institutional Review Boards: Including the Comité de Protection des Personnes Ile-de-France II, the McGill University Health Centre Research Institute, the Academic Medical Centre of Amsterdam The Netherlands,the Ethics Committee of the Second University of Naples, the Bioethics Committee of the University of Barcelona, the Ethics Committee of Rotterdam and Nijmegen (MEC-2001-23, MEC-2005-371, and MEC-2010-359), the Baylor College of Medicine Institutional Review Board, the Ethics committee of the Medical Faculty of University of Tubingen and the Zhongshan Ophthalmic Center of Sun Yat-sen University.

**Table 1:** Cohorts of patients affected with various retinal dystrophies screened for *RD3* mutations.

	Origin	Number patients	Ethnicity	Screening methodology
**LCA (n = 574)**	Central America	10	Mexican	1
	North America	513	Unspecified mixed	1,2
	China	78	Han Chinese	1
	Europe			
	France	190	Mixed population	1
	Germany	1	Turkish origin	
	Italy	90	Italian	1
	Spain	5	Spanish	1
**EOSRD (n = 96)**	Europe (The Netherlands)	96	Mostly Dutch patients	1
**RD** * **(n = 182)**	Europe (Italy)	150	Italian	1
	China	11+21**	Han Chinese	1

* Retinal dystrophies including cone/cone-rod dystrophies and unclassified RDs;

** Patients with blindness or severe visual deficiency but ERG data unavailable.1 Direct sequencing and 2 SCCP screening of the two coding exons and intron-exon boundaries.

### Screening of *RD3*

Samples were collected and screened for *RD3* mutations independently. Patients of all cohorts but one were screened by direct sequencing using intronic primers designed to amplify the two RD3 coding exons and intron-exon boundaries. The 313 LCA probands of the University of Iowa Carver College cohort were screened by single strand conformation using as controls 151 normal individuals. PCR, direct sequencing and SSCP conditions, as well as primer sequences are available on request.

A variant was predicted to be pathogenic when it showed familial cosegregation with the disease and absence in control individuals and when it was predicted to be damaging by Align DGVD, Polyphen-2, SIFT, SpliceSiteFinder-like, MaxEntScan, NNSPLICE and Human Splicing Finder available through the Alamut Interpretation Software 2.0.

### Linkage disequilibrium analysis

Haplotype analysis was performed using 4/5 families segregating the c.112C>T mutation of the *RD3* gene. Microsatellite markers distributed over a 12.6 Megabases (Mb) interval spanning the *RD3* locus on chromosome 1q32.2-q41 (according to the UCSC Genome Browser GRCh37/hg19 assembly) were used for haplotype analysis. Microsatellite markers included (from telomere to centromere): AFMB347YA5 (D1S2782), AFM281YG1 (D1S471), AFM310VB1 (D1S491), AFM108YA3 (D1S205), AFM179XG5 (D1S414) AFMC011YD5 (D1S2810), AFMB342YG1 (D1S2780), AFM203ZB6 (D1S425), AFMA127WB5 (D1S505), AFM297XC1 (D1S2827), AFM058XG9 (D1S2880). Amplified fragments were electrophoresed on an automatic sequencer (ABI 3100, Applied Biosystems, Foster City, CA) and analyzed using the GeneScan Analysis 3.7 and Genotyper softwares. For each marker, the heterozygote frequency and the size range of alleles were either available from Genethon Linkage Map [36]. The size of the alleles carried by the members of families NEM1, NEM2, NEM3 and NEM4 was determined relative to the reference DNA sequence obtained from the Centre d'Etude du Polymorphisme Humain (CEPH).

The degree of linkage disequilibrium and the estimation of the age of the mutation were obtained as described previously [37].

## Results

### *RD3* mutations, changes of uncertain pathogenicity and polymorphisms

Three novel mutations were detected in seven apparently unrelated LCA families (Table 2). Two out of the three mutations affect codon c.136–138 (exon 2), which encodes the glutamic acid at position 46. The first one consists of a 2 bp deletion expected to result in the substitution of the glutamic acid into an alanine and the apparition of a premature stop codon, 26 amino-acids downstream (c.137–138delAG; p.E46Afs*83). Sequence analysis indicates that the microdeletion is not expected to alter the splicing of the messenger RNA (Human Splicing Finder, SpliceSiteFinder-like, MaxEntScan, NNSplice). The second change consists of a G to T transversion expected to change the glutamic acid at position 46 by a signal of termination of the translation (c.136G>T, p.E46*) and/or to create a cryptic donor-splice site which use would lead to the production of a 141-aa protein lacking aa 46–99 and thus the C terminus of the predicted coiled coil domain (Human Splicing Finder score 71.2 vs exon 2 acceptor site 96.7). The third mutation occurred on the nt112–113 CpG doublet (c.112C>T). It is expected to change the arginine at position 38 by a signal of termination of the translation (p.R38*) and/or to create a cryptic donor-splice site which use would result in the production of a 132-aa protein lacking amino acids 38–99 including the putative coiled-coil domain (Human Splicing Finder, SpliceSiteFinder-like, MaxEntScan). All three mutations are expected to truncate the RD3 protein. It is thus possible that the mutant mRNAs may be prone to nonsense-mediated decay. It is interesting to note that the E46 position may be hypermutable (2 different mutations affecting the codon).

**Table 2:** *RD3* mutations and related retinal phenotype.

Position	Mutation type	Mutation	Predicted effect on protein	Family/Patient& gender/age (age at examination if different)	Country of origin	Light behavior	Refraction	Visual acuity	Fundus Aspect	Remarks
**Exon 2**	Nonsense	c.112C>T	p.R38*	NEM1/II1F/2.5	Morocco	Photoaversion	−1.50 LE;−1.50 RE (measured without cycloplegic)	NA	Macular rearrangement, With abnormal macular pigmentation with starshape Diffuse salt and pepper aspect, thin vessels	Nystagmus since birth, no ocular pursuit, altered pupillary reflexes
				NEM2/II1F/31	Morocco	Photoaversion	NA	LP LRE	NA	Nystagmus since birth altered pupillary reflexes, Keratoconus
				NEM2/II2F/27		Photoaversion	NA	LP LRE	NA	Nystagmus since birth, altered pupillary reflexes
				NEM3/II2F/12	Lebanon	Photoaversion	+2 RE;+2.75 LE	LP LE; HM RE	Salt and pepper retina, thin vessels, marked macular atrophy	Nystagmus at birth, DOSF
				NEM3/II3F/9(8)		NA	+1 LRE	CF RE;HM LE	NA	
				NEM3/II4M/2(1)		NA	NA	HM LRE	NA	
				NEM4/II1F/22(4)	Turkey	Photoaversion	+4 LRE	LP LRE	Macular rearrangement, thin retinal vessels, dull retina with no pigmentary migrations	
				NEM4/II2F/10(3)	Turkey	Photoaversion	+3 LRE	LP LRE	Macular rearrangement, thin retinal vessels, dull retina with no pigmentary migrations	
				F16338/LCA59-2-94F/17	Turkey	Photoaversion	NA	LP LRE	Macular atrophy, RPE atrophy with salt and pepper aspect, some bone-spicule pigment in the mid periphery	Nystagmus since birth, altered papillary reflexes
**Exon 2**	Deletion	c.137–138delAG	p.E46Afs83*	NEM5/II1F/21(4)	Algeria	Photoaversion	+4 LRE	LP LRE	Macular rearrangement (4 yrs), thin retinal vessels, dull retina with no pigmentary migrations	Nystagmus since birth, no ocular pursuit, altered pupillary reflexes
**Exon 2**	Nonsense	c.136G>T	p.E46*	FJC/F/4(3)	Mexico	No photoaversion, No nyctalopia	+1.25 LRE	HM LRE	Macular atrophy, attenuated retinal vessels, abnormal RPE in the mid-periphery, optic disk pallor	Nystagmus since birth, DOSF

LE: left eye; RE: right eye; LP: light perception; HM: hand movements; CF: counting fingers; DOSF: digito-ocular signs of Franceschetti; NA: not available.

All three mutations segregated with the disease in families and were neither identified in controls nor reported elsewhere.

**Figure 1:**
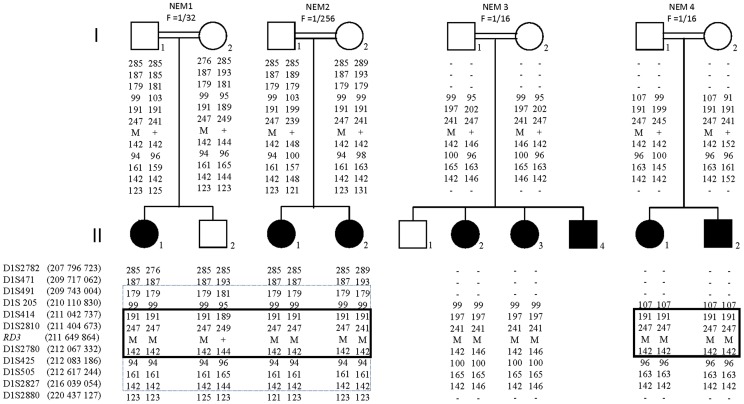
Pedigree structure of the 4/5 families segregating the c.112C>T mutation of the *RD3* gene with haplotypes reconstruction for informative markers on chromosome 1q32.2-q41. Black circles (women) and squares (men) indicate affected members. Chromosomal positions of SNP and microsatellite markers are indicated in Mega base pairs (Mb) according to the UCSC Genome Browser GRCh37/hg19 assembly. Common haplotypes in families NEM1, NEM2 and in families NEM1, NEM2, NEM3 are shared with dotted and solid lines, respectively. NA not available.

The c.112C>T mutation was shared by 5/7 of the families segregating *RD3* mutations. Indirect studies at the *RD3* locus were performed on 4/5 families indicate that the two Moroccan families and the Turkish family share a small 1 Mb-long common haplotype that differed in the Libanese family (Figure 1). Haplotype analyses and Bayesian calculations allowed estimating that the founder mutation might have occurred 100–150 generations ago.

In addition to *RD3* mutations, we identified a A to T transversion at position 146 of the cDNA resulting into the substitution of an asparagine into an isoleucine at position 49 of the protein (c.146A>T; N49I). This variant that changes a basic amino acid into an uncharged residue is predicted to be deleterious by the SIFT, Polyphen-2 and AlignGCVD programs. The change was identified neither in 200 control individuals nor in databases listing SNPs. This change was identified in a heterozygous state in an isolated case born to non-consanguineous parents (Table 3).

**Table 3:** *RD3* Changes of uncertain pathogenicity.

Position	Mutation type	Mutation	Predicted effect on protein	Conservation of the mutant aa	Predicted effect (SIFT, Polyphen-2, AlignGCVD)	Remarks
Exon 2	Missense	c.146A>T	p.N49I	Low	Deleterious	1/200 patient (European descent, single heterozygosity)

Finally, 13 polymorphic variants were identified, 3/13 of which were not reported elsewhere (Table 4). All three changes (2/3 non-conservative) were predicted to be benign by the Alamut Interpretation Software.

**Table 4:** *RD3* Coding SNPs.

Position	Polymorphism type	Change	Predicted effect on protein	Rs numnber	Reference
Exon 2	Non-conservative	c.16T>C	p.W6R	rs35649846	dbSNP
Exon 2	Non-conservative	c.69G>C	p.E23D	rs34422496	dbSNP
Exon 2	Non-conservative	c.83C>T	p.T28M	rs61740157	dbSNP
Exon 2	Conservative	c.84G>A	p.T28T	rs61740158	dbSNP
Exon 2	Non-conservative	c.101C>T	p.T34M	-	This study
Exon 2	Non-conservative	c.103G>A	p.G35R	rs34049451	dbSNP
Exon 2	Non-conservative	c.139C>T	p.R47C	rs34049451	dbSNP
Exon 2	Non-conservative	c.202C>T	p.R68W	-	Friedman et al. 2006
Exon 2	Conservative	c.235T>C	p.L79L	rs35937732	dbSNP
Exon 3	Conservative	c.498C>T	p.I166I	-	This study
Exon 3	Non-conservative	c.500G>A	p.R167K	rs74782684	dbSNP
Exon 3	Non-conservative	c.511G>A	p.E171K	-	This study
Exon 3	Non-conservative	c.584A>T	p.D195V	-	Friedman et al. 2006; This study

Novel non-conservative changes were regarded as polymorphisms when they did not segregate with the disease in patients and/or were found in control individuals. References: 1:http://www.ncbi.nlm.nih.gov/SNP/; 2: Friedman et al. 2006. All Sequence change data are based on the RefSeq cDNA sequence NM_183059.2.

### Phenotype of patients harboring *RD3* mutations

**Figure 2:**
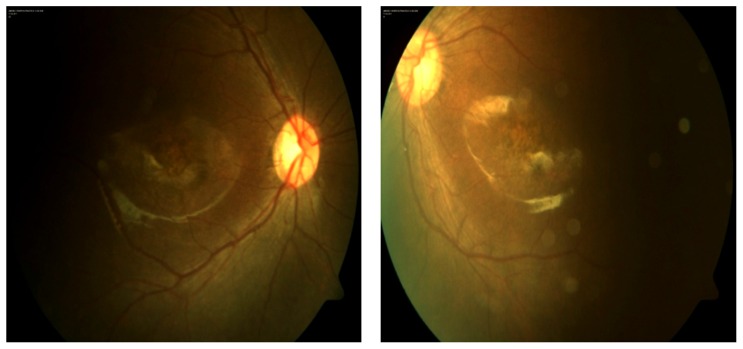
Fundus photographs of the affected person FJC/F (**Table 2A**). The proband has poor vision since birth with nystagmus and atrophic lesions in the macular area.

The natural history of the disease and clinical data available of patients harboring *RD3* mutations were reviewed (11 patients in 7 families; mean age  = 10.2 yrs; age range 1–31 yrs, Table 2). Patients consistently presented with congenital nystagmus, low vision, sluggish pupillary reflexes, absence of ocular pursuit since birth, early-onset and long-lasting digito-ocular signs of Franceschetti, photoaversion (one exception) and mild to moderate hyperopia. Visual acuity, when measurable, was reduced to counting fingers, hand-movements or light perception. Reduced visual acuity hindered recording color vision and visual field (Table 2). Fundus consistently showed dull retina with salt and pepper aspect, thin retinal vessels and an early macular rearrangement (maculopathy, Figure 2). Spectral Domain OCT imaging was available in the Tübingen case. The photoreceptor layer was missing, the inner segment/outer segment border was diminished and an increased backscatter from the choroid was observed due to RPE-atrophy. On the other hand, inner retinal layers seemed to show a rather preserved structure. Fundus autofluorescence (FAF) was severely reduced, a proper image could not be taken.

## Discussion

Until this report, the implication of *RD3* in retinal dystrophies was based on the following three studies: homozygosity for truncating mutations in two consanguineous LCA families [2/59; 21,35], two naturally occurring models of autosomal recessive EOSRD, the *retinal degeneration 3* (*rd3*) mouse [21], [38] and the *rod-cone dystrophy 2* (*rcd2*) collie [39]–[40]. The identification in our report of three mutations expected to truncate the protein in seven additional families gives strong support to the role of *RD3* in LCA. Yet, the mutation rate obtained in a very large cohort of LCA cases originating worldwide (7/574 LCA cases excluding other LCA genes) and consistent identification of homozygous mutations in consanguineous families [21,35, present study], support the very low frequency of heterozygous *RD3* carriers in the general population.

*RD3* encodes an evolutionary conserved basic protein of 195 amino acids that is preferentially expressed in the retina [41]. In the mouse, it exhibits increasing expression through early postnatal development. In adulthood, it is highly expressed in cone and rod outer segments where it co-localizes with retinal-specific guanylate cyclases, GC1 and GC2, respectively. In the cones, RD3 has been shown to mediate the export of GC1 from the endoplasmic reticulum to endosomal vesicles with the C terminus of GC1 being required for RD3 binding [21], [41]–[42]. The loss of GC1 and GC2 in the photoreceptors of the *rd3* mouse which express a truncated unstable rd3 protein resulting from the p.R107* mutation, is consistent with a role of the protein in the trafficking of GC2 in rods as well. As a consequence, like the double GC1/GC2 knock-out, the complete inactivation of both *rd3* alleles is expected to preclude cGMP synthesis in both rod and cone outer segments and to hinder recovery of the dark state after light stimulation [21], [42]–[43]. Consistently, the retina of the animal develops normally but both rod and cone photoreceptors start degenerating by postnatal day 14 - *i.e.* when eyes open – and the degeneration is almost complete by the age of 2–4 months [21], [38], [44]. Similarly, the *rd3* frameshift mutation (p.Pro139Alafs*) of the *rcd2* collie [40] is likely to produce an unstable, non-functional rd3 protein leading to the loss of GC expression and trafficking. The retina of homozygous *rcd2* dogs develops almost normally, by the age of 2–2.5 months, the outer segments of both rods and cones are completely lost, with rods degenerating faster than cones [45].

Interestingly, these data which support a common mechanism of photoreceptor degeneration in *rd3* and GC double knock-out mice [43] are consistent with the phenotypic overlap between *LCA12* and *LCA1*. Indeed, the review of the natural history and clinical data of our patients and that of the two original *RD3* families [21], [35] delineate a homogeneous phenotype unambiguously consistent with the diagnosis of congenital and dramatically severe cone-rod dystrophy (LCA type I) originally described in *LCA1* patients harboring *GUGY2D* mutations [3].

*RD3* mutations identified thus far are expected to truncate the RD3 protein [21,35, present study]. It is tempting to hypothesize that similar to several other LCA genes, mutations with a milder effect on the protein structure and/or function may be responsible for milder phenotypes; for recent reviews see [1], [2]. However, none of the unrelated patients affected by various retinal dystrophies of later-onset and/or severity in our cohort (n = 278) and the other two reported series (n = 907) [21], [35], [40] exhibited disease-causing *RD3* mutations. This finding is consistent with the LCA type I-specific involvement of *RD3* mutations. Indeed, of the LCA genes, most genes involved in EOSRD and RP account for LCA type II [1].

Until now, four LCA genes account for congenital cone-rod dystrophy (LCA type I): *GUCY2D* [3], *RPGRIP1* [21], *CEP290* [46] and *RD3* [present study]. Refraction may represent a distinctive feature in LCA type I since patients with *GUCY2D* mutations consistently have high hypermetropia (>+7) with frequent enophthalmia [3], [25] whereas those who harbor *RPGRIP1, CEP290* or *RD3* mutations present with lower or no hyperopia ([25], [46], present study). On the other hand, several population-specific mutations were identified in LCA type I genes. In Mediterranean countries where *CEP290* mutations are particularly uncommon [46]–[47], high frequencies of *GUCY2D* mutations resulting from founder effects have been reported [25], [37], [48]. With the present report, *RD3* mutations were identified in nine consanguineous families, 6/9 of which originate from Mediterranean countries. Five out of the six families harbored the c.112C>T mutation. Considering, the very low frequency of this mutation in populations with different genetic backgrounds, it is unlikely that it results from the recurrent deamination of the cytosine of the nt112–113 CpG doublet [49]. Besides, indirect studies at the *RD3* locus were consistent with the transmission of an ancient founder mutation that occurred in the Mediterranean region. The prevalence of a RD3 mutation in Mediterranean countries allows us to improve the diagnostic flowchart drawn to direct the molecular analysis of LCA type I genes previously reported [1], [2]. In LCA type I patients originating from this region with moderate or no hyperopia, *RD3* should be screened for mutations first, before *GUCY2D*, *RPGRIP1* and *CEP290*.

With respect to genetic counseling, our data that support a dramatically low RD3 carrier frequency in the general population indicate that the genetic risk in the case of the union between an affected person harboring *RD3* mutations and an individual of the general population with no family history of LCA is negligible. Unions between affected persons with LCA and or between LCA and RP patients are not uncommon. The identification of *RD3* mutations in one spouse must prompt the search for *RD3* mutations, only when the other spouse is affected with LCA type I. Indeed, if the spouse is affected with LCA type II or autosomal recessive RP of later-onset, the genetic counseling can straightaway be reassuring.

The comprehensive survey of *RD3* mutations in large cohorts of patients accurately phenotyped by referent clinicians has major clinical implications. Most LCA genes have demonstrated a preferential or specific retinal expression [1], [2]. Consistently, their alterations account for isolated forms of the disease. Yet, increasing numbers of disease-causing genes display greater and more wide-spread patterns of expression including *CEP290* which mutations are either responsible for non-syndromic LCA or a range of syndromic forms of the disease; for review see [32]. On the other hand, mutations in the *IQCB1* gene at the *NPHP5* locus have been shown to cause a form of Senior-Loken syndrome characterized by a congenital retinal dystrophy associated with a renal dysfunction of highly variable age of onset (1^st^ to 5^th^ decade) [27], [28], [33]. These recent findings support the view that the identification of the disease gene may be a valuable prognostic tool in young individuals affected with LCA. With regard to *RD3*, it is worth noting that none of the patients reported here whose ages ranged from 2 to 31 had renal failure, neurological symptoms or intellectual disability. Owing to the retina-specific pattern of expression of *RD3*, it is likely that the disease may remain restricted to this tissue. In these patients, anxiogenic and expensive extraocular explorations must not be systematically prescribed.

The recovery of the dark state in the retina through GC-mediated increase in cytoplasmic cGMP concentration in photoreceptor outer segments is *a sine qua none* condition to maintain living and functional rod and cone cells. Our knowledge regarding the mechanisms of this complex process - which alteration accounts for ∼17% of LCA cases - are incomplete. In particular, most of the molecular players and disease mechanisms of light-driven trafficking between the inner and outer segments of photoreceptors are unknown. Interestingly, although *RD3* alterations are quite uncommon, their description in man and animal models unraveled a crucial protein for GC expression and trafficking in photoreceptors. Further studies designed to identify RD3 partners will hopefully contribute to the deciphering of mechanisms for dark-state recovery and will hopefully allow uncovering some of the yet unidentified LCA causing genes.

Finally, it is worth remembering that the success of gene replacement and drug therapy to restore vision in naturally occurring *RPE65−/−* Briard dogs has paved the way for the development of gene and drug therapy for a broad range of eye disorders and made large dogs the species of choice for these developments; for review see [50]. In this context, although *RD3* mutations are uncommon causes of LCA, the availability of naturally occurring *rd3* animal models, among which a large dog (collie), it is likely that gene and drug therapy protocols will be developed to treat patients with *RD3* mutations.

## References

[1] Kaplan J (2008) Leber congenital amaurosis: from darkness to spotlight. Ophthalmic Genet 29: 92–98. 18766987 10.1080/13816810802232768

[2] den Hollander AI, Roepman R, Koenekoop RK, Cremers FP (2008) Leber congenital amaurosis: genes, proteins and disease mechanisms. Prog Retin Eye Res 27: 391–419 Review. 18632300 10.1016/j.preteyeres.2008.05.003

[3] Perrault I, Rozet JM, Ghazi I, Leowski C, Bonnemaison M, et al (1999) Different functional outcome of RetGC1 and RPE65 gene mutations in Leber congenital amaurosis. Am J Hum Genet 64: 1225–1228. 10090910 10.1086/302335 PMC1377849

[4] Perrault I, Rozet JM, Calvas P, Gerber S, Camuzat A, et al (1996) Retinal-specific guanylate cyclase gene mutations in Leber's congenital amaurosis. Nat Genet 14: 461–464. 8944027 10.1038/ng1296-461

[5] Marlhens F, Bareil C, Griffoin JM, Zrenner E, Amalric P, et al (1997) Mutations in RPE65 cause Leber's congenital amaurosis. Nat Genet 17: 139–141. 9326927 10.1038/ng1097-139

[6] Wang H, den Hollander AI, Moayedi Y, Abulimiti A, Li Y, et al (2009) Mutations in SPATA7 cause Leber congenital amaurosis and juvenile retinitis pigmentosa. Am J Hum Genet 84: 380–387. 19268277 10.1016/j.ajhg.2009.02.005 PMC2668010

[7] Sohocki MM, Bowne SJ, Sullivan LS, Blackshaw S, Cepko CL, et al (2000) Mutations in a new photoreceptor-pineal gene on 17p cause Leber congenital amaurosis. Nat Genet 24: 79–83. 10615133 10.1038/71732 PMC2581448

[8] den Hollander AI, Koenekoop RK, Mohamed MD, Arts HH, Boldt K, et al (2007) Mutations in LCA5, encoding the ciliary protein lebercilin, cause Leber congenital amaurosis. Nat Genet 39: 889–895. 17546029 10.1038/ng2066

[9] Dryja TP, Adams SM, Grimsby JL, McGee TL, Hong DH, et al (2001) Null RPGRIP1 alleles in patients with Leber congenital amaurosis. Am J Hum Genet 68: 1295–1298. 11283794 10.1086/320113 PMC1226111

[10] Gerber S, Perrault I, Hanein S, Barbet F, Ducroq D, et al (2001) Complete exon-intron structure of the RPGR-interacting protein (RPGRIP1) gene allows the identification of mutations underlying Leber congenital amaurosis. Eur J Hum Genet 9: 561–71. 11528500 10.1038/sj.ejhg.5200689

[11] Freund CL, Wang QL, Chen S, Muskat BL, Wiles CD, et al (1998) De novo mutations in the CRX homeobox gene associated with Leber congenital amaurosis. Nat Genet 18: 311–312. 9537410 10.1038/ng0498-311

[12] Swaroop A, Wang QL, Wu W, Cook J, Coats C, et al (1999) Leber congenital amaurosis caused by a homozygous mutation (R90W) in the homeodomain of the retinal transcription factor CRX: direct evidence for the involvement of CRX in the development of photoreceptor function. Hum Mol Genet 8: 299–305. 9931337 10.1093/hmg/8.2.299

[13] den Hollander AI, Heckenlively JR, van den Born LI, de Kok YJ, van der Velde-Visser SD, et al (2001) Leber congenital amaurosis and retinitis pigmentosa with Coats-like exudative vasculopathy are associated with mutations in the crumbs homologue 1 (CRB1) gene. Am J Hum Genet 69: 198–203. 11389483 10.1086/321263 PMC1226034

[14] Keen TJ, Mohamed MD, McKibbin M, Rashid Y, Jafri H, et al (2003) Identification of a locus (LCA9) for Leber's congenital amaurosis on chromosome 1p36. Eur J Hum Genet 11: 420–423. 12734549 10.1038/sj.ejhg.5200981

[15] Chiang PW, Wang J, Chen Y, Fu Q, Zhong J, et al (2012) Exome sequencing identifies NMNAT1 mutations as a cause of Leber congenital amaurosis.Nat Genet. Jul 29;44 (9) 972–4. 10.1038/ng.2370 22842231

[16] Koenekoop RK, Wang H, Majewski J, Wang X, Lopez I, et al (2012) Mutations in NMNAT1 cause Leber congenital amaurosis and identify a new disease pathway for retinal degeneration. Nat Genet. Jul 29;44 (9) 1035–9. 10.1038/ng.2356 PMC3657614 22842230

[17] Perrault I, Hanein S, Zanlonghi X, Serre V, Nicouleau M, et al (2012) Mutations in NMNAT1 cause Leber congenital amaurosis with early-onset severe macular and optic atrophy. Nat Genet. Jul 29;44 (9) 975–7. 10.1038/ng.2357 22842229

[18] Falk MJ, Zhang Q, Nakamaru-Ogiso E, Kannabiran C, Fonseca-Kelly Z, et al (2012) NMNAT1 mutations cause Leber congenital amaurosis. Nat Genet. Jul 29;44 (9) 1040–5. 10.1038/ng.2361 PMC3454532 22842227

[19] den Hollander AI, Koenekoop RK, Yzer S, Lopez I, Arends ML, et al (2006) Mutations in the CEP290 (NPHP6) gene are a frequent cause of Leber congenital amaurosis. Am J Hum Genet 79: 556–561. 16909394 10.1086/507318 PMC1559533

[20] Bowne SJ, Sullivan LS, Mortimer SE, Hedstrom L, Zhu J, et al (2006) Spectrum and frequency of mutations in IMPDH1 associated with autosomal dominant retinitis pigmentosa and leber congenital amaurosis. Invest Ophthalmol Vis Sci 47: 34–42. 16384941 10.1167/iovs.05-0868 PMC2581444

[21] Friedman JS, Chang B, Kannabiran C, Chakarova C, Singh HP, et al (2006) Premature truncation of a novel protein, RD3, exhibiting subnuclear localization is associated with retinal degeneration. Am J Hum Genet 79:1059–70. Erratum in: Am J Hum Genet 2007 80: 388. 10.1086/510021 PMC1698706 17186464

[22] Janecke AR, Thompson DA, Utermann G, Becker C, Hübner CA, et al (2004) Mutations in RDH12 encoding a photoreceptor cell retinol dehydrogenase cause childhood-onset severe retinal dystrophy. Nat Genet 36: 850–854. 15258582 10.1038/ng1394

[23] Perrault I, Hanein S, Gerber S, Barbet F, Ducroq D, et al (2004) Retinal dehydrogenase 12 (RDH12) mutations in leber congenital amaurosis. Am J Hum Genet 75: 639–646. 15322982 10.1086/424889 PMC1182050

[24] Thompson DA, Li Y, McHenry CL, Carlson TJ, Ding X, et al (2001) Mutations in the gene encoding lecithin retinol acyltransferase are associated with early-onset severe retinal dystrophy. Nat Genet 28: 123–124. 11381255 10.1038/88828

[25] Hanein S, Perrault I, Gerber S, Tanguy G, Barbet F, et al (2004) Leber congenital amaurosis: comprehensive survey of the genetic heterogeneity, refinement of the clinical definition, and genotype-phenotype correlations as a strategy for molecular diagnosis. Hum Mutat 23: 306–317. 15024725 10.1002/humu.20010

[26] Sergouniotis PI, Davidson AE, Mackay DS, Li Z, Yang X, et al (2011) Recessive mutations in KCNJ13, encoding an inwardly rectifying potassium channel subunit, cause leber congenital amaurosis. Am J Hum Genet 89: 183–190. 21763485 10.1016/j.ajhg.2011.06.002 PMC3135807

[27] Estrada-Cuzcano A, Koenekoop RK, Coppieters F, Kohl S, Lopez I, et al (2011) IQCB1 mutations in patients with leber congenital amaurosis. Invest Ophthalmol Vis Sci 52: 834–839. 20881296 10.1167/iovs.10-5221

[28] Stone EM, Cideciyan AV, Aleman TS, Scheetz TE, Sumaroka A, et al (2011) Variations in NPHP5 in patients with nonsyndromic Leber congenital amaurosis and Senior-Loken syndrome. Arch Ophthalmol 129: 81–87. 21220633 10.1001/archophthalmol.2010.330 PMC3952880

[29] Gal A, Li Y, Thompson DA, Weir J, Orth U, Jacobson SG, et al (2000) Mutations in MERTK, the human orthologue of the RCS rat retinal dystrophy gene, cause retinitis pigmentosa. Nature Genet 26: 270–271. 11062461 10.1038/81555

[30] Sohocki MM, Sullivan LS, Mintz-Hittner HA, Birch D, Heckenlively JR, et al (1998) A range of clinical phenotypes associated with mutations in CRX, a photoreceptor transcription-factor gene. Am J Hum Genet 63: 1307–1315. 9792858 10.1086/302101 PMC1377541

[31] Perrault I, Hanein S, Gerber S, Barbet F, Dufier JL, et al (2003) Evidence of autosomal dominant Leber congenital amaurosis (LCA) underlain by a CRX heterozygous null allele. J Med Genet 40: e90. 12843339 10.1136/jmg.40.7.e90 PMC1735514

[32] Coppieters F, Lefever S, Leroy BP, De Baere E (2010) CEP290, a gene with many faces: mutation overview and presentation of CEP290base. Hum Mutat 31: 1097–1108. 20690115 10.1002/humu.21337

[33] Otto EA, Loeys B, Khanna H, Hellemans J, Sudbrak R, et al (2005) Nephrocystin-5, a ciliary IQ domain protein, is mutated in Senior-Loken syndrome and interacts with RPGR and calmodulin. Nat Genet 37: 282–288. 15723066 10.1038/ng1520

[34] Chung DC, Traboulsi EI (2009) Leber congenital amaurosis: clinical correlations with genotypes, gene therapy trials update, and future directions. J AAPOS 13: 587–592. 20006823 10.1016/j.jaapos.2009.10.004

[35] Preising MN, Hausotter-Will N, Solbach MC, Friedburg C, Rüschendorf F, et al (2012) Mutations in RD3 Are Associated with an Extremely Rare and Severe Form of Early Onset Retinal Dystrophy. Invest Ophthalmol Vis Sci. Jun 8;53 (7) 3463–72. 10.1167/iovs.12-9519 PMC3390007 22531706

[36] Dib C, Fauré S, Fizames C, Samson D, Drouot N, et al (1996) A comprehensive genetic map of the human genome based on 5,264 microsatellites. Nature 380: 152–1544.9. 8600387 10.1038/380152a0

[37] Hanein S, Perrault I, Gerber S, Delphin N, Benezra D, et al (2008) Population history and infrequent mutations: how old is a rare mutation? GUCY2D as a worked example. Eur J Hum Genet 16: 115–123. 17684531 10.1038/sj.ejhg.5201905

[38] Chang B, Heckenlively JR, Hawes NL, Roderick TH (1993) New mouse primary retinal degeneration (rd-3). Genomics 16: 45–49. 8486383 10.1006/geno.1993.1138

[39] Wolf ED, Vainisi SJ, Santos-Anderson R (1978) Rod-cone dysplasia in the collie. J Am Vet Med Assoc 173: 1331–1333. 730609

[40] Kukekova AV, Goldstein O, Johnson JL, Richardson MA, Pearce-Kelling SE, et al (2009) Canine RD3 mutation establishes rod-cone dysplasia type 2 (rcd2) as ortholog of human and murine rd3. Mamm Genome 20: 109–123. 19130129 10.1007/s00335-008-9163-4 PMC2652121

[41] Lavorgna G, Lestingi M, Ziviello C, Testa F, Simonelli F, et al (2003) Identification and characterization of C1orf36, a transcript highly expressed in photoreceptor cells, and mutation analysis in retinitis pigmentosa. Biochem Biophys Res Commun 308: 414–421. 12914764 10.1016/s0006-291x(03)01410-4

[42] Azadi S, Molday LL, Molday RS (2010) RD3, the protein associated with Leber congenital amaurosis type 12, is required for guanylate cyclase trafficking in photoreceptor cells. Proc Natl Acad Sci U S A 107: 21158–21163. 21078983 10.1073/pnas.1010460107 PMC3000275

[43] Baehr W, Karan S, Maeda T, Luo DG, Li S, et al (2007) The function of guanylate cyclase 1 and guanylate cyclase 2 in rod and cone photoreceptors. J Biol Chem 282: 8837–8847. 17255100 10.1074/jbc.M610369200 PMC2043484

[44] Linberg KA, Fariss RN, Heckenlively JR, Farber DB, Fisher SK (2005) Morphological characterization of the retinal degeneration in three strains of mice carrying the rd-3 mutation. Vis Neurosci 22: 721–734. 16469183 10.1017/S0952523805226044

[45] Santos-Anderson RM, Tso MO, Wolf ED (1980) An inherited retinopathy in collies. A light and electron microscopic study. Invest Ophthalmol Vis Sci 19: 1281–1294. 7429765

[46] Perrault I, Delphin N, Hanein S, Gerber S, Dufier JL, et al (2007) Spectrum of NPHP6/CEP290 mutations in Leber congenital amaurosis and delineation of the associated phenotype. Hum Mutat 28: 416. 10.1002/humu.9485 17345604

[47] Aboussair N, Berahou A, Perrault I, Elalaoui SC, Megzari A, et al (2010) First North African observation of Leber congenital amaurosis secondary to CEP290 gene mutation. J Fr Ophtalmol 33: 117.e1–5. 20056295 10.1016/j.jfo.2009.11.009

[48] Hanein S, Perrault I, Olsen P, Lopponen T, Hietala M, et al (2002) Evidence of a founder effect for the RETGC1 (GUCY2D) 2943DelG mutation in Leber congenital amaurosis pedigrees of Finnish origin. Hum Mutat 20: 322–3. 10.1002/humu.9067 12325031

[49] Glass JL, Thompson RF, Khulan B, Figueroa ME, Olivier EN, et al (2007) CG dinucleotide clustering is a species-specific property of the genome. Nucleic Acids Res 35: 6798–807. 17932072 10.1093/nar/gkm489 PMC2175314

[50] Cideciyan AV (2010) Leber congenital amaurosis due to RPE65 mutations and its treatment with gene therapy. Prog Retin Eye Res 29: 398–427. 20399883 10.1016/j.preteyeres.2010.04.002 PMC2903652

